# Semiquantitative ^18^F-FDG PET/CT in monitoring glucocorticoid response of immunoglobulin G4-related effusive constrictive pericarditis: a case report

**DOI:** 10.1186/s12872-024-03797-z

**Published:** 2024-02-22

**Authors:** Soo Yeon An, Byung Joo Sun

**Affiliations:** 1 Department of Cardiology, Chungnam National University Hospital, Moonhwa-lo 282, Jung-gu, Daejeon, 35015 Republic of Korea; 2 School of Medicine, Department of Medical Sciences, Institute of Cardiology, Chungnam National University, Daejeon, Republic of Korea; 3 Division of Cardiology, Asan Medical Center, University of Ulsan College of Medicine, Seoul, Republic of Korea

**Keywords:** Immunoglobulin G4-related disease, Effusive constrictive pericarditis, ^18^F-FDG PET/CT multimodality imaging, Glucocorticoids

## Abstract

**Background:**

Immunoglobulin G4 (IgG4)-related effusive constrictive pericarditis (ECP) is a rare manifestation of IgG4-related disease (IgG4-RD). It can lead to persistent pericardial fibrosis, resulting in cardiac tamponade, diastolic dysfunction, and heart failure. Glucocorticoids are the primary treatment for effectively reducing inflammation and preventing fibrosis. However, guidelines for monitoring treatment response are lacking and tapering glucocorticoid therapy for specific target organs remains a challenge. Recent studies on IgG4-RD have demonstrated that semiquantitative measurements of fluorine-18 fluorodeoxyglucose (^18^F-FDG) uptake in the main involved organs in positron emission tomography/computed tomography (PET/CT) scanning are correlated to disease activity. We present a case of IgG4-related ECP to demonstrate the usefulness of ^18^F-FDG PET/CT for diagnosing and treatment follow-up of IgG4-related ECP.

**Case presentation:**

Herein, a 66-year-old woman diagnosed with IgG4-related ECP presented with breathlessness, leg swelling, rales, and fever. Laboratory tests revealed markedly elevated levels of C-reactive protein, and transthoracic echocardiography revealed constrictive physiology with effusion. High IgG4 levels suggested an immune-related pathogenesis, while viral and malignant causes were excluded. Subsequent pericardial biopsy revealed lymphocyte and plasma cell infiltration in the pericardium, confirming the diagnosis of IgG4-related ECP. ^18^F-FDG PET/CT revealed increased uptake of ^18^F-FDG in the pericardium, indicating isolated cardiac involvement of IgG4-RD. Treatment with prednisolone and colchicine led to a rapid improvement in the patient’s condition within a few weeks. Follow-up imaging with ^18^F-FDG PET/CT after 3 months revealed reduced inflammation and improved constrictive physiology on echocardiography, leading to successful tapering of the prednisolone dose and discontinuation of colchicine.

**Conclusion:**

The rarity of IgG4-related ECP and possibility of multiorgan involvement in IgG4-RD necessitates a comprehensive diagnostic approach and personalized management. This case report highlights the usefulness of ^18^F-FDG PET/CT in the diagnosis and treatment follow-up of isolated pericardial involvement in IgG4-RD.

## Background

Immunoglobulin G4 (IgG4)-related disease (IgG4-RD) is a systemic fibroinflammatory disease characterized by the dense infiltration of IgG4-positive plasma cells in affected tissues [1]. Effusive constrictive pericarditis (ECP) is a rare manifestation of IgG4-RD, contributing to pericardiac fibrosis [2]. Cardiac involvement of IgG4-RD is rare, but it can result in life-threatening cardiac complications if cardiac inflammation and fibrosis are left untreated [3]. Glucocorticoids are the main treatment for IgG4-RD, but there is a lack of consensus on how to monitor and taper glucocorticoid treatment for specific target organs [4]. Recent studies have demonstrated that fluorine-18 fluorodeoxyglucose (^18^F-FDG) positron emission tomography/computed tomography (PET/CT) scanning is beneficial for treatment follow-up of IgG4-related cardiovascular diseases, providing functional information about disease activity [5].

Herein, we present a rare case of IgG4-related ECP in a 66-year-old woman demonstrating the effectiveness of ^18^F-FDG PET/CT to help clinical management of IgG4-ECP.

## Case presentation

A 66-year-old woman was admitted to hospital complaining of shortness of breath and edema of the lower extremities for two weeks. The patient had no personal or family history of similar diseases.

The patient had an initial blood pressure of 120/70 mmHg, a heart rate of 90 beats per min, a respiratory rate of 22 breaths per min, and a low-grade fever (37.5 °C). Physical examination revealed rales at the base of the left lung and pitting edema in both lower extremities. Initial electrocardiographic examination showed a normal sinus rhythm and a low R-wave voltage throughout the limb leads. The complete blood count showed leukocytosis (white blood cell count of 12.77 × 10^3^/µL). She had a C-reactive protein level of 14.7 mg/dL (normal range < 0.5 mg/dL) and an N-terminal pro-B-type natriuretic peptide level of 823.9 pg/mL (normal range < 125 pg/mL).

**Fig. 1:**
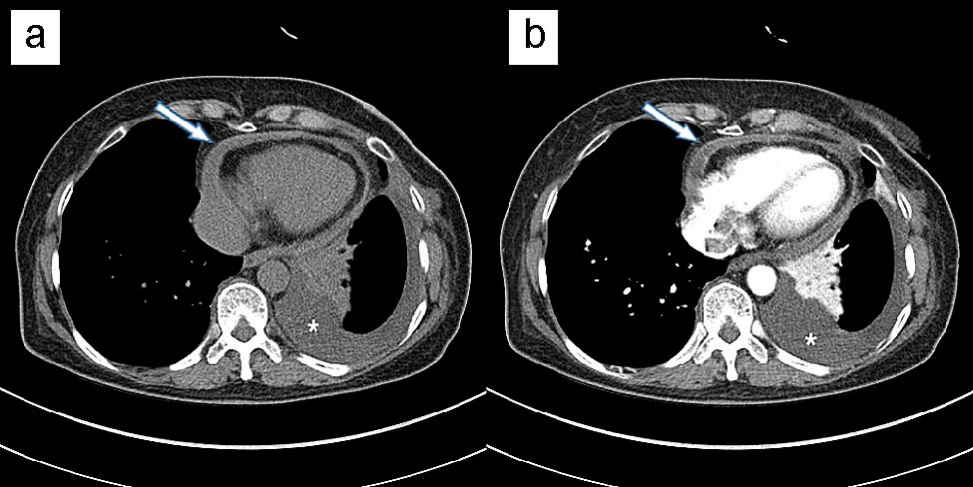
Chest computed tomography (CT) scan. (**a**) Non-enhanced image of the chest CT scan showing left-sided pleural effusion (asterisk) and pericardial thickening of up to 12 mm (white arrow). (**b**) Contrast-enhanced image showing pericardial effusions (white arrow) with pericardial rim enhancement suggesting effusive pericarditis. CT: computed tomography

Contrast-enhanced chest computed tomography (CT) scans revealed left-sided pleural and pericardial effusions (Fig. 1a), and the pericardium was thickened by up to 12 mm with contrast enhancement in the peripheral rims of the pericardium (Fig. 1b).

**Fig. 2:**
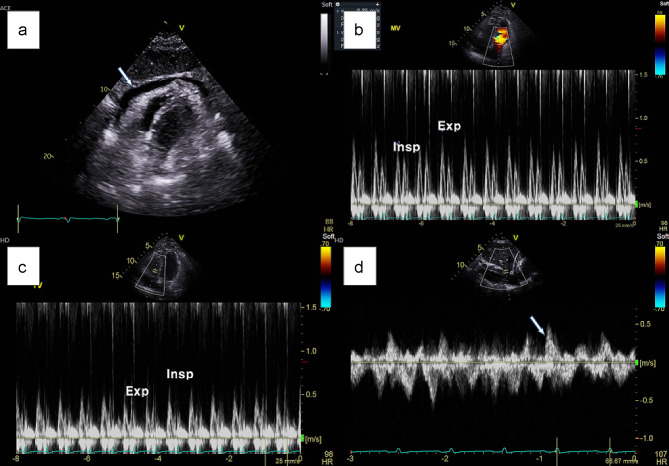
Transthoracic echocardiogram showing constrictive physiology. (**a**) Pericardial thickening and organized pericardial effusion are observed anterior to the right ventricular wall (arrow). (**b**) Mitral inflow velocity varies with respiration. (**c**) Tricuspid valve velocity varies with respiration. (**d**) Hepatic vein diastolic reversal flow with expiration is observed on pulse wave Doppler imaging (arrow)

Transthoracic echocardiography revealed a septal bounce, indicating interventricular dependence and pericardial thickening anterior to the right ventricular wall (Fig. 2a). The medial e’ velocity (11.4 cm/s) was greater than the lateral left ventricular e’ velocity (8.9 cm/s), and the mitral inflow and tricuspid valve velocity varied with respiration (Fig. 2b and c). Hepatic vein diastolic reversal flow during expiration was also observed on pulsed-wave Doppler (Fig. 2d) and color Doppler imaging. The inferior vena cava was dilated and plethoric, indicating elevated right atrial pressure. The cardiac chambers were not dilated, and the right and left ventricular systolic functions were normal. Based on these findings, ECP was suspected.

Further laboratory and imaging examinations were performed to exclude infectious or malignant etiologies of ECP. Pleural effusion analysis revealed a lymphocyte-dominant transudate effusion with no evidence of malignancy or infection. Gram staining, cultures of pleural effusion, acid-fast bacilli staining, *Mycobacterium tuberculosis* real-time polymerase chain reaction, and other common viral tests showed negative results, and the adenosine deaminase test yielded normal values. Additional laboratory tests were performed to assess the possibility of undiagnosed malignancies or autoimmune diseases. The results of autoantibody tests were negative, but the serum IgG4 level was 2,550 mg/dL (normal range: 30–2010 mg/dL). Hence, IgG4-RD with pericardial involvement was suspected.

**Fig. 3:**
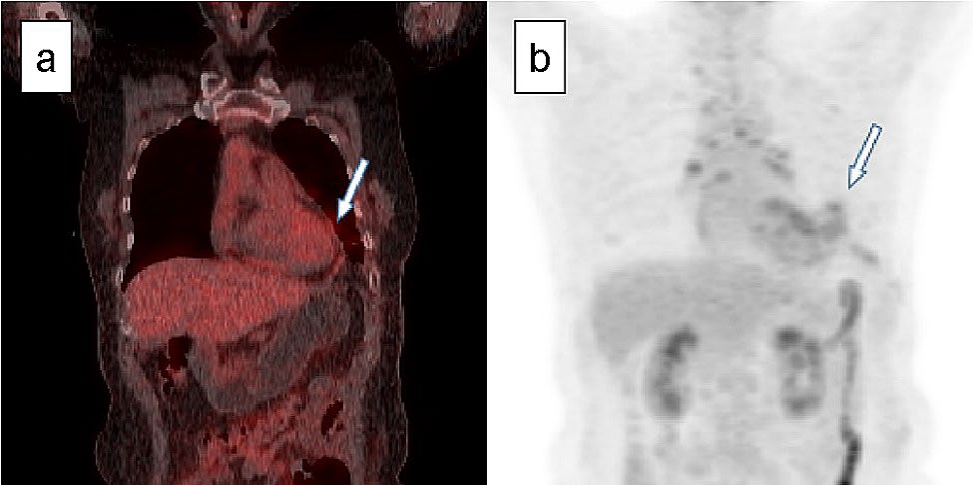
^18^F-FDG-PET/CT image before glucocorticoid treatment. (**a**) ^18^F-FDG-PET/CT scan showing increased ^18^F-FDG uptake in the pericardium (SUVmax = 2.9), indicating active inflammation (arrow). (**b**) ^18^F-FDG uptake in the pericardium (arrow) in the PET image. ^18^F-FDG-PET/CT: fluorine-18 fluorodeoxyglucose -positron emission tomography/computed tomography, SUVmax: maximum standardized uptake value

The patient underwent an ^18^F-FDG PET/CT scanning, revealing patchy uptake of ^18^F-FDG in the pericardium, indicating isolated pericardial involvement of IgG4-RD (Fig. 3a and b). Semiquantitative measurement of ^18^F-FDG uptake and the maximum standardized uptake value (SUVmax) of the pericardium was 2.9, indicating active pericardial inflammation.

**Fig. 4:**
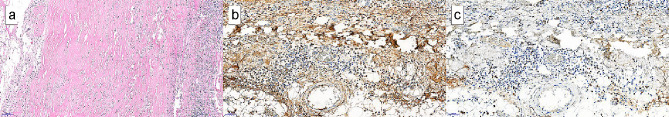
Pericardial biopsy. (**a**) The tissue section of the pericardium showing diffuse pericardial thickening with dense plasma cell infiltration. (**b**) Immunohistochemistry of IgG. (**c**) Immunohistochemistry of IgG4 demonstrated > 50 IgG4-positive cells per high-power field with an IgG4/IgG ratio of > 20%, consistent with IgG4-related disease. IgG: immunoglobulin G, IgG4: immunoglobulin G4

A pericardial biopsy was performed via the pericardial window to confirm the diagnosis. Pericardial thickening and concurrent serous effusion were observed during surgery. A histopathological examination of the biopsy specimen revealed a thickened fibrocollagenous pericardium with dense plasma cell infiltration (Fig. 4a). Immunohistochemistry revealed > 50 IgG4-positive plasma cells per high-power field with an IgG4/IgG ratio of > 20%. The final diagnosis was IgG4-related ECP (Fig. 4b and c).

The patient was initially treated with furosemide and colchicine. Once the definite diagnosis of IgG4-related ECP was established, prednisolone at a dose of 40 mg/day was initiated. After initiating the glucocorticoid regimen, the patient remained symptom-free for a week. The patient was discharged without complications after 1 month. The prednisolone dose was decreased to 30 mg/day with a gradual tapering plan.

**Fig. 5:**
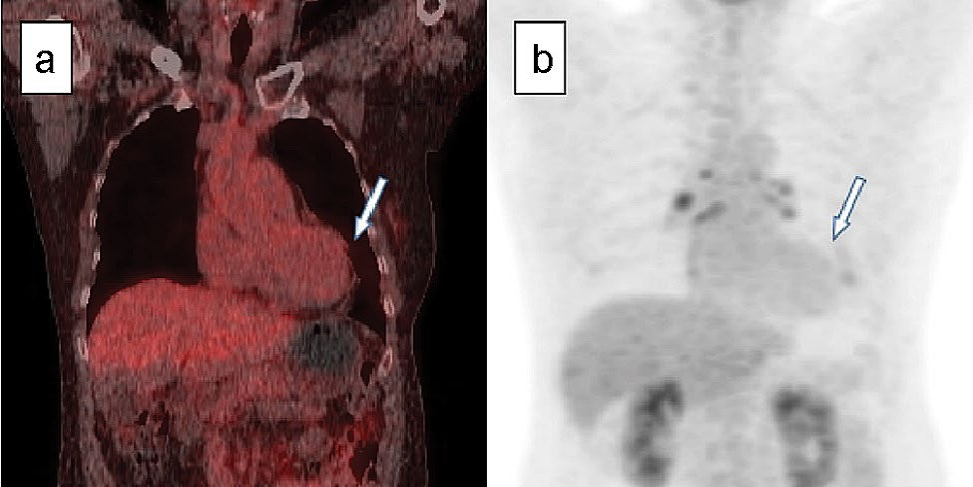
Follow-up ^18^F-FDG-PET/CT image. (**a**) A repeat ^18^F-FDG-PET/CT scan was performed after 3 months of glucocorticoid treatment, and the pericardial inflammation markedly improved with a decreased ^18^F-FDG uptake (SUVmax = 1.5) in the pericardium (arrow). (**b**) ^18^F-FDG uptake in the pericardium (arrow) in PET image. ^18^F-FDG-PET/CT: fluorine-18 fluorodeoxyglucose -positron emission tomography/computed tomography, SUVmax: maximum standardized uptake value

The patient was maintained on prednisolone and colchicine therapy for the next 3 months. Repeat echocardiography revealed improvement in pericardial thickening and constrictive physiology. Follow-up ^18^F-FDG PET/CT imaging confirmed a reduction in pericardial inflammation (Fig. 5a and b), with markedly decreased ^18^F-FDG uptake in the pericardium (SUVmax = 1.5). Based on these findings, colchicine was discontinued, and prednisolone was tapered to a maintenance dose of 10 mg/day. The IgG4 level decreased to 1,360 mg/dL, and the patient remained stable without disease recurrence over a 24-month follow-up period.

## Discussion and conclusions

IgG4-RD is a fibroinflammatory condition with multiorgan involvement. ECP, although rare, is a potentially life-threatening condition requiring clinical acumen for diagnosis [6]. Definite diagnosis of IgG4-RD can be made according to the 2020 Revised Comprehensive Diagnostic criteria incorporating clinical, serological, and pathological indicators [7]. Thorough exclusion of other potential diagnoses is a cautious approach, which may delay definite diagnosis and initiation of treatment. Although a cautious approach will ensure appropriate management aligning with best practices in clinical decision-making, delaying treatment might endanger patients, especially with cardiac involvement of IgG4-RD with unstable hemodynamics. Employing ^18^F-FDG PET/CT scanning in the early diagnostic stage might be an optimal strategy for patients with IgG4-RD with cardiac involvement. ^18^F-FDG PET/CT allows for early detection of inflammation that may precede structural changes [8]. ^18^F-FDG PET/CT is a valuable tool in monitoring IgG4-RD, wherein multiple organ systems might be involved. It is highly sensitive and allows for the evaluation of multiorgan involvement in a single examination, an advantage that echography does not possess [9]. ^18^F-FDG PET/CT can also aid in the differential diagnosis of IgG4-RD, guide biopsy, and help monitor treatment response [10]. Furthermore, it can help detect characteristic ^18^F-FDG accumulation in the affected organs, aiding in the diagnosis of IgG4-RD and the assessment of treatment effects [11]. Despite some limitations, ^18^F-FDG PET/CT has demonstrated utility in assessing IgG4-RD both at initial evaluation and after therapy [12]. In our patient, echocardiography detected constrictive physiology of the heart. Once we suspected IgG4-RD as indicated by an elevated serum IgG4 level, we performed ^18^F-FDG PET/CT, which helped to pinpoint the inflammation in the pericardium and confidently exclude the involvement of other organs. Accordingly, the patient underwent the surgical biopsy of the pericardium, and a definite diagnosis of IgG4-related ECP was made before initiating glucocorticoid treatment.

We also utilized ^18^F-FDG PET/CT to monitor treatment response over long-term follow-up. Currently, guidelines for monitoring treatment response are lacking and appropriately tapering glucocorticoid therapy remains challenging. However, ^18^F-FDG PET/CT scanning is evidently useful for diagnosing and monitoring IgG4-related cardiovascular diseases [5]. The decrease in ^18^F-FDG uptake after glucocorticoid therapy, indicated by a decreased SUVmax value, significantly correlated with disease activity [13]. The disappearance of ^18^F-FDG uptake reflects a positive response to treatment, thus validating its use in monitoring treatment response in the long-term follow-up of IgG4-RD. The semiquantitative measurements of ^18^F-FDG uptake and SUVmax values of the involved organs have been positively correlated with disease activity in previous studies [14]. However, several factors can affect the SUV measurements, including reconstruction iterations [15], blood glucose levels, diabetes, insulin treatment and obesity [16], and image reconstruction parameters [17, 18]. These factors can lead to variations in the SUV measurements, which in turn can impact the interpretation of PET/CT results. The influence of patient weight change on the uptake value in the region of interest in ^18^F-FDG PET/CT scans is a complex issue. Therefore, it is important to standardize the ^18^F-FDG PET/CT procedures to ensure consistent SUV measurements [19]. Nevertheless, the reproducibility of SUV measurements has been demonstrated despite these challenges [20]. Particularly, treatment response can be monitored by the disappearance of ^18^F-FDG uptake, indicating successful treatment of IgG4-RD. In our case, follow-up ^18^F-FDG PET/CT scanning along with echocardiography after 3 months of treatment sufficiently aided the clinical decision to taper down the dose of prednisolone. In real-world practice, individual variations in treatment responses and disease progression may affect generalizability. In the present case, the patient’s body weight decreased from 59 kg to 53 kg over 3 months; which could indeed have impacted the SUV in PET/CT imaging as the SUV is calculated by normalizing the tissue radioactivity concentration to the administered dose and patient’s weight. As such, changes in body weight can affect the SUV. In the present case, the initial edema and overestimated body weight could have likely resulted in a lower calculated SUV, as the same amount of radioactivity would have been normalized over a higher body weight. Conversely, with the subsequent weight loss (presumably with the resolution of edema), the SUV could have appeared falsely elevated due to the normalization of radioactivity over a reduced body weight. This phenomenon is particularly relevant in conditions with changes in body composition, such as edema or cachexia. Therefore, when interpreting the PET/CT findings, it is crucial to consider changes in body weight, especially in conditions that can lead to significant fluid shifts. The lack of established guidelines for ^18^F-FDG PET/CT monitoring warrants caution when interpreting the imaging results.

Besides recognizing various factors that can affect SUV measurements, a useful method can increase the accuracy of the assessment of pericardial inflammation: Horn et al. [21] evaluated the reliability of ^18^F-FDG PET/CT imaging biomarkers in classifying early treatment response in patients with non-small cell lung cancer. They investigated the variability across observers, scanners, and reconstruction algorithms in the context of biologically adaptive radiation therapy. Importantly, they could provide new, effective ways to improve the diagnostic accuracy of ^18^F-FDG PET/CT in pericarditis evaluation. Their method involves minimizing the normal myocardial uptake of ^18^F-FDG in ^18^F-FDG PET/CT scans, which enhances the specificity in detecting inflammatory processes, such as pericarditis. The suppression of the physiological myocardial ^18^F-FDG uptake makes the pathological uptake patterns indicative of pericarditis more discernible. Their method could particularly be useful in conditions such as IgG4-ECP, wherein accurate assessment of inflammation is crucial. Moreover, the suppression of physiological uptake allows for clearer differentiation between normal and abnormal metabolic activities, leading to a more precise localization of inflammation and improved disease management.

This case highlights the significance of a combined clinical and imaging approach and individualized treatment in IgG4-related ECP. When the patient’s response to glucocorticoid treatment was evaluated, ^18^F-FDG uptake measurements proved helpful in determining treatment efficacy and planning a tapering strategy. Establishing the appropriate timeline for the initiation of glucocorticoid treatment and optimal duration of glucocorticoid therapy is necessary in the future studies.

## Data Availability

The datasets used and/or analyzed during the current study available from the corresponding author on reasonable request.

## Abbreviations

CT: Computed tomography
TTE: Transthoracic echocardiography
ECP: Effusive constrictive pericarditis
IgG4: Immunoglobulin G4
IgG4-RD: IgG4-related disease
^18^F-FDG: fluorine-18 fluorodeoxyglucose
PET/CT: Positron emission tomography/computed tomography
SUV: Standardized uptake value
SUVmax: Maximum standardized uptake value
